# A 12-year follow up survey of childhood blindness at schools for the blind in Cambodia

**DOI:** 10.1186/s12886-024-03285-0

**Published:** 2024-02-13

**Authors:** Thomas Rogerson, Sith Sam Ath, Ngy Meng, Robert Casson

**Affiliations:** 1 Sight For All Foundation, Adelaide, South Australia Australia; 2 Department of Ophthalmology, The Royal Adelaide Hospital, Port Road, 5000 Adelaide, South Australia Australia; 3 Discipline of Ophthalmology and Visual Science, University of Adelaide, Adelaide, South Australia Australia; 4 National Programme for Eye Health, Ministry of Health, Phnom Penh, Cambodia

**Keywords:** Childhood blindness, Cambodia, Child, Schools for the Blind, Ophthalmic Public Health, Epidemiology, Paediatric Ophthalmology

## Abstract

**Background:**

Cambodia is a low-income country in South East Asia with a population of 15.5 million people of whom 4.9 million (38%) are under the age of 16. The causes of childhood blindness in Cambodia have not been investigated since the first survey of schools for the blind done in 2009 by our group. Given the large demographic and economic shifts in Cambodia since 2009 it is important to determine if these causes have changed in order to ensure intervention programmes are appropriately targeted. The purpose of the present study is to investigate the prevalence of causes of childhood blindness at schools for the blind in Cambodia.

**Methods:**

Students between the ages of 5 and 16 years who were attending schools for the blind in Cambodia were examined by a consultant paediatric ophthalmologist and had clinical photographs taken. Distance visual acuity was measured using a logMAR tumbling E chart and the WHO definitions of blindness and severe visual impairment were used. The examining ophthalmologist recorded the anatomical site and aetiology of vision loss using the WHO Prevention of Blindness eye examination record for children. Collected data were compared to a previous survey from 2009.

**Results:**

Data from 73 students were included for analysis. The most common anatomical location of abnormality causing vision loss was the cornea (*n* = 20, 33.9%) followed by the lens and retina (*n* = 11, 18.64% each). Hereditary factors (*n* = 29, 49.15%) and childhood diseases (*n* = 27, 45.76%) were the most common aetiological causes of childhood blindness. The majority (71.19%) of childhood blindness was avoidable. The present study did not demonstrate 0a significant difference in the causes of childhood blindness compared to 2009.

**Conclusions:**

Corneal pathologies continue to represent the most common cause of vision loss amongst the surveyed population and the majority of causes of childhood blindness continue to be avoidable. These findings will facilitate the development of evidence-based targeted interventional programmes in Cambodia.

## Background

Childhood blindness is an important amalgamation of disease entities that affect 1.4 million children world-wide, account for the same number of “blind-years” as age-related cataracts and often result in life-long limitations in social and economic engagement for the individual afflicted [1]. Understanding the distribution of causes of childhood blindness is a vital prerequisite to developing appropriately targeted, evidence-based public health policies and interventional programs; however, in many instances the disparate root causes of childhood blindness are incompletely understood. In addition, the root causes of childhood blindness such as vitamin A deficiency and measles are closely interlinked with causes of childhood mortality and there is a significant systemic and mortality benefit to the prevention of these illnesses [2]. Indeed so strong is this association, that one of the best ways to estimate the prevalence of childhood blindness is to use a country’s under-5 childhood mortality rate [3].

Despite the importance of identifying these causes there is still a relative paucity of information regarding the specific aetiological breakdown of childhood blindness in many developing world countries, including Cambodia, where the present study was conducted. Although the “gold standard” of these data would be large population-based surveys, the relatively low prevalence of childhood blindness means that the study population of such a project is enormous and the cost prohibitive. To try to mitigate the size of the study population and associated cost, “key informants” have been used by other researchers to identify children who may be visually impaired in the community and then refer them on to a centralised study team for full examination. This methodology is also imperfect because it is reliant on an original assessment by a variety of different lay members of the community and there remain significant costs to the family associated with transport for assessment and treatment. Hence, one commonly used approach, which the current study employs, is to survey children attending a school or schools for the blind and use this sample to draw conclusions regarding the general population. This is consistent with recommendations from the World Health Organisation (WHO) and internationally accepted norms [4].

Cambodia is a low-income country in South East Asia bordering Laos, Vietnam and Thailand. It has a population of 15.5 million people of whom 4.9 million (38%) are under the age of 16 [5]. In the absence of official data, we estimate that there are 3.2 million children aged between 5 and 16 years and that the prevalence of childhood blindness in Cambodia is 0.8 per 1000 children. This would suggest that there are approximately 2500 blind children in Cambodia. Of these, only 96 are enrolled at a school for the blind. The reasons for this are multitudinous. There is only limited access to ophthalmic care in many rural areas of Cambodia which leads to an under-recognition of blind children; there is no national registry of the blind in Cambodia which makes the care of blind children highly de-centralised; transport within Cambodia is often cost and time-prohibitive which means that rural families are unable to access one of the four schools for the blind; and schools for the blind are unable to accept students who have a disability in addition to blindness e.g. hearing loss or intellectual impairment. As a general principle, these limitations are not specific to Cambodia, but are reflective of the current status quo in developing countries. Notwithstanding these limitations, previous authors have demonstrated that with the exception of slightly higher prevalence of cortical visual impairment amongst children in the community, data obtained from surveys of schools for the blind are representative of causes of blindness in the community [6–8].

Within Cambodia, although a general shift towards urbanisation was demonstrated between the 2008 and 2019 censuses, Cambodian society remains relatively agrarian with only 39.4% of the population living in an urban setting (increased from 19.5% in 2008) [9]. Although it has experienced a period of rapid growth in the last two decades, the Cambodian populace continues to battle poverty and all of its associated adverse health outcomes; data for the most recent year from the World Bank shows that GDP per capita was $1625.20 USD in 2021 [10].

The present study is the 12 year follow up to the first Cambodian childhood blindness study (CCBS1), and was undertaken to provide insights into how the prevalence of different causes of childhood blindness has changed in Cambodia and how future interventions can be tailored to meet demand. Our group conducted the only previous study investigating the prevalence of causes of childhood blindness in Cambodia in 2009 the data from which have gone on to be used by the Cambodian Ministry of Health (MOH), Cambodian National Programme for Eye Health as well as Non-Government Organisations (NGOs) such as Sight For All Foundation to plan interventions to reduce the prevalence of preventable blindness in children [11].

## Materials and methods

Children attending all four schools for the blind in Cambodia for whom consent to participate had been provided were examined by study investigators in December of 2021. In line with previously established protocols for surveys of schools for the blind, only children between the ages of 5 and 18 years old are included in our presented data. Relevant past medical and ophthalmic history was obtained from medical and school records as well as staff and children. Students underwent a complete ophthalmic examination by one of two Cambodian consultant paediatric ophthalmologists and a clinical diagnosis was formulated based upon these findings. Distance visual acuity (VA) was measured using a logMAR tumbling E chart; the WHO definitions of blindness (defined as VA < 3/60 in the better eye) and severe visual impairment (VA < 6/60 to 3/60 in the better eye) were used [12]. A child was considered to have functional vision if they could navigate between two chairs placed one meter apart. Tonometry was performed using Icare (IC100) (Revenio Group, Vantaa, Finland). The anterior segment was assessed using slit lamp biomicroscopy and the posterior segment was evaluated using indirect ophthalmoscopy with dilatation of the pupil. Children who had an improvement in the distance visual acuity with pinhole underwent subjective refraction. Where refraction improved the acuity, distance spectacles were ordered and dispensed.

The examining ophthalmologist recorded the anatomical site and aetiology of the vision loss. The anatomical location of blindness was defined as the primary location of the abnormality responsible for vision loss in each child. Where causes of vision loss differed between two eyes, the most preventable or treatable cause of blindness was selected, and where both causes were equally avoidable, the most recent cause of vision loss was recorded. These methods are consistent with CCBS1.

The need for optical, surgical or medical interventions was recorded and visual potential assessed. When required, arrangements were made for these interventions. All students participating in the study had ophthalmic photographs taken and these were reviewed and the diagnosis was cross-checked by an experienced Australian ophthalmologist (RJC). In cases where the photographic appearance did not appear consistent with the clinical diagnosis the child’s case was reviewed and a consensus diagnosis was reached between study members.

All findings were recorded using the WHO Prevention of Blindness (PBL) eye examination record for children [13]. Data were transcribed and collated using Microsoft Excel (Seattle, WA, USA) and analysed using SPSS v27.0 (Chicago, Il, USA). Descriptive statistics are reported below and data from the present study were compared to those from 2009 using Chi-squared tests for each individual location of blindness. *P*-values reported were derived from Pearson’s coefficient for corneal, lenticular, retinal and whole globe causes of blindness. Yate’s continuity correction was employed for the less prevalent causes of childhood blindness including causes arising from the optic nerve, refractive error and uveal tissue.

### Ethics approval

The present study was approved by the Cambodian National Ethics Committee for Health Research. This study adhered to the tenets of the Declaration of Helsinki. Approvals to visit these schools were obtained from the Cambodian Ministry of Health and Cambodian Ministry of Education Youth and Sport.

## Results

Of the 96 students who were enrolled at the 4 schools for the blind in Cambodia, 73 were examined in December of 2021. Of these, 47 were found to be blind and 12 were severely visually impaired. Reported demographic and disease data are for these students only (Table 1). Of the 59 blind or severely visually impaired students the median student age was 13 (IQR 11–15); 15 students (25.42%) reported a family history of blindness, a parental history of consanguinity was present in 11(18.64%) and 52 (88.13%) reported an age of onset prior to one year of age. There were 16 children who had undergone previous surgical procedures the most common of which was cataract extraction with or without intraocular lens insertion (*n* = 8, 13.56%) followed by corneal graft in 3 students (5.08%), enucleation/evisceration in 3 (5.08%) students, retinopexy in 1 student (1.69%), and trabeculectomy in 1 (1.69%).

Using the standardised classification system endorsed by the WHO, the main anatomical location of abnormality causing vision loss was the cornea (*n* = 20, 33.9%) followed by the lens (*n* = 11, 18.64%), retina (*n* = 11, 18.64%), whole globe (*n* = 10, 16.94%), optic nerve (*n* = 5, 8.47%) and refractive error (*n* = 2, 3.39%) (Table 2) [13].

Using a self-reporting methodology with corroboration with medical records at the schools for the blind, 52 students (88.1%) were found to have become blind prior to one year of age. The largest cause amongst this group was corneal blindness (19 students, 36.5%) followed by disorders of the lens and retina (10 students each, 19.2%). The causes of blindness amongst this age group did not differ significantly compared to causes of blindness amongst the entire study population.

Hereditary factors and childhood diseases were the most common causes of childhood blindness, accounting for nearly half of all cases each: 29 (49.2%) and 27 students (45.8%), respectively (Table 3). The aetiology of blindness was attributable to intrauterine and perinatal factors in one student each and was unknown in one student.

The cause of childhood blindness reported in the present study was considered to be avoidable in 42 students (71.2%). Of these, 23 cases were preventable (39.0%) and 19 were potentially treatable (32.2%) (Table 4). Almost half of all avoidable blindness, 42.9%, was attributable to corneal pathologies (*n* = 18), while 11 children had lenticular causes of treatable blindness, five children had glaucoma (four congenital and one neovascular) and only one child was blind due to retinopathy of prematurity (ROP).

**Table 1:** Demographic Data on blind children attending schools for the blind

Demographic category	Males No. (%)	Female No. (%)	Total No. (%)
School			
Krousar Thmey, Siem Reap	10 (16.9)	7 (11.9)	17 (28.8)
Krousar Thmey, Battambang	3 (5.1)	2 (3.4)	5 (8.5)
Krousar Thmey, Phnom Penh	16 (27.1)	11 (18.6)	27 (45.8)
Krousar Thmey Kampong Cham	6 (10.2)	4 (6.8)	10 (16.9)
Age			
0–5	0	0	0
6–10	5 (8.5)	10 (16.9)	15 (25.4)
11–15	24 (40.7)	13 (22.0)	37 (62.7)
16–18	6 (10.2)	1 (1.7)	7 (11.9)
Level of visual impairment			
Blind	27 (45.8)	20 (33.9)	47 (79.7)
Severe Visual Impairment	8 (13.6)	4 (6.8)	12 (20.3)
Other disability			
Yes	2 (3.4)	0	2 (3.4)
No	33 (55.9)	24 (40.7)	57 (96.6)
Family History			
Yes	9 (15.3)	6 (10.2)	15 (25.4)
No	26 (44.1)	18	44 (74.6)
History of Consanguinity			
Yes	10 (16.9)	1 (1.7)	11 (18.6)
No	25 (42.4)	23 (39.0)	48 (81.4)
Age of Onset			
Prior to 1 year of age	32 (54.2)	24 (40.7)	52 (88.1)
1–5 years of age	1 (1.7)	0	1 (1.7)
5 to 16 years of age	2 (3.4)	0	2 (3.4)
Unknown	1 (1.7)	3 (5.1)	4 (6.8)
Previous surgery			
Yes	10 (16.9)	7 (11.9)	17 (28.8)
No	25 (42.4)	17 (28.8)	42 (71.2)
Total	35 (59.3)	24 (40.7)	59 (100)

Legend: Demographic data, disaggregated by sex, for all students with childhood blindness or severe visual impairment

**Table 2:** Causes of blindness and Severe Visual Impairment by anatomical location

Causes of blindness and SVI by anatomical location	Male No (%)	Female No. (%)	Total No. (%)
**Cornea**	**11 (18.6)**	**9 (15.3)**	**20 (33.9)**
Band Keratopathy	1 (1.7)	1 (1.7)	2 (3.4)
Congenital Hereditary Endothelial Dystrophy	1 (1.7)	0	1 (1.7)
Corneal Scar	6 (10.2)	5 (8.5)	11 (18.6)
Sclerocornea	2 (3.4)	1 (1.7)	3 (5.1)
Staphyloma	1 (1.7)	2 (3.4)	3 (5.1)
**Lens**	**5 (8.5)**	**6 (10.2)**	**11 (18.6)**
Cataract	5 (8.5)	5 (8.5)	10 (16.9)
aphakia	0	1 (1.7)	1 (1.7)
**Retina**	**9 (15.3)**	**2 (3.4)**	**11 (18.6)**
Macula Scar?infective	1 (1.7)	0	1 (1.7)
PHPV	1 (1.7)	0	1 (1.7)
Retinal dystrophy	5 (8.5)	1 (1.7)	6 (10.2)
Retinitis pigmentosa	1 (1.7)	0	1 (1.7)
Retinoblastoma	0	1 (1.7)	1 (1.7)
Retinopathy of prematurity	1 (1.7)	0	1 (1.7)
**Optic Nerve**	**3 (5.1)**	**2 (3.4)**	**5 (8.5)**
Lebers Optic Nerve atrophy	1 (1.7)	0	1 (1.7)
Optic Nerve atrophy	2 (3.4)	1 (1.7)	3 (5.1)
Optic Nerve Hypoplasia	0	1 (1.7)	1 (1.7)
**Whole Globe**	**6 (10.2)**	**4 (6.8)**	**10 (16.9)**
Ocular Vit A deficiency	0	1 (1.7)	1 (1.7)
Phthisis	3 (5.1)	1 (1.7)	4 (6.8)
Congenital glaucoma	2 (3.4)	2 (3.4)	4 (6.8)
Neovascular glaucoma	1 (1.7)	0	1 (1.7)
**Refractive Error**	1 (1.7)	1 (1.7)	**2 (3.4)**
**Total**	**35 (59.3)**	**24 (40.7)**	**59 (100)**

Legend: Data disaggregated by sex depicting the breakdown of causes of Cambodian childhood blindness by anatomical location

**Table 3:** Causes of blindness by aetiology

Aetiological factor	Male No. (%)	Female No (%)	Total No. (%)
Hereditary disease	18 (30.5)	11 (18.6)	29 (49.2)
Intrauterine	1 (1.7)	0	1 (1.7)
Perinatal/Neonatal	1 (1.7)	0	1 (1.7)
Postnatal/infancy/childhood	14 (23.7)	13 (22.0)	27 (45.8)
Unknown	1 (1.7)	0	1 (1.7)
**Total**	**35**	**24**	**59 (100)**

Legend: Data disaggregated by sex depicting the breakdown of causes of Cambodian childhood blindness and severe visual impairment by aetiological factor

**Table 4:** Avoidable causes of blindness

	Male No. (%)	Female No. (%)	Total No. (%)
**Avoidable causes of blindness**	**21 (35.6)**	**19 (32.2)**	**40 (67.8)**
**Preventable**	**11 (18.6)**	**10 (16.9)**	**21 (35.6)**
Band Keratopathy	1 (1.7)	1 (1.7)	2 (3.4)
Corneal scar/staphyloma (EG from measles, Vitamin A deficiency, ophthalmia neonatorum etc.)	7 (11.9)	7 (11.9)	14 (23.7)
Vitamin A def	0	1 (1.7)	1 (1.7)
Phthisis	3 (5.1)	1 (1.7	4 (6.8)
**Treatable**	**10 (16.9)**	**9 (15.3)**	**19 (32.2)**
Cataract	5 (8.5)	4 (6.8)	9 (15.3)
Pseudophakia/Aphakia	0	2 (3.4)	2 (3.4)
Glaucoma	3 (5.1)	2 (3.4)	5 (8.5)
Refractive error	1 (1.7)	1 (1.7)	2 (3.4)
ROP	1 (1.7)	0	1 (1.7)
**Unavoidable**	**14 (23.7)**	**5 (8.5)**	**19 (32.2)**
Retinal Dystrophies	6	1 (1.7)	10 (16.9)
Congenital Hereditary Endothelial Dystrophy	1 (1.7)	0	1 (1.7)
PHPV	1 (1.7)	0	1 (1.7)
Retinoblastoma	0	1 (1.7)	1 (1.7)
Macula Scar (vertical transmission)	1 (1.7)	0	1 (1.7)
Optic nerve atrophy/hypoplasia	3 (5.1)	2 (3.4)	5 (8.5)
Sclerocornea	2 (3.4)	1 (1.7)	3 (5.1)
**Total**	**35**	**24**	**59 100**

Legend: Data disaggregated by sex depicting the breakdown of causes of childhood blindness and severe visual impairment into preventable, treatable and unavoidable categories amongst Cambodian children in 2021

**Table 5:** Comparison of 2021 and 2009 data

Location	CCBS1 (2009)	CCBS2 (2021)	Chi-Squared *p*-value
Cornea	16	20	0.330
Lens	17	11	0.253
Retina	13	11	0.749
Whole Globe	11	10	0.908
Optic Nerve	2	5	0.397
Refractive Error	1	2	0.527
Uvea	2	0	0.498

Legend: Number of children in each anatomical group with severe visual impairment or blindness

**Fig. 1:**
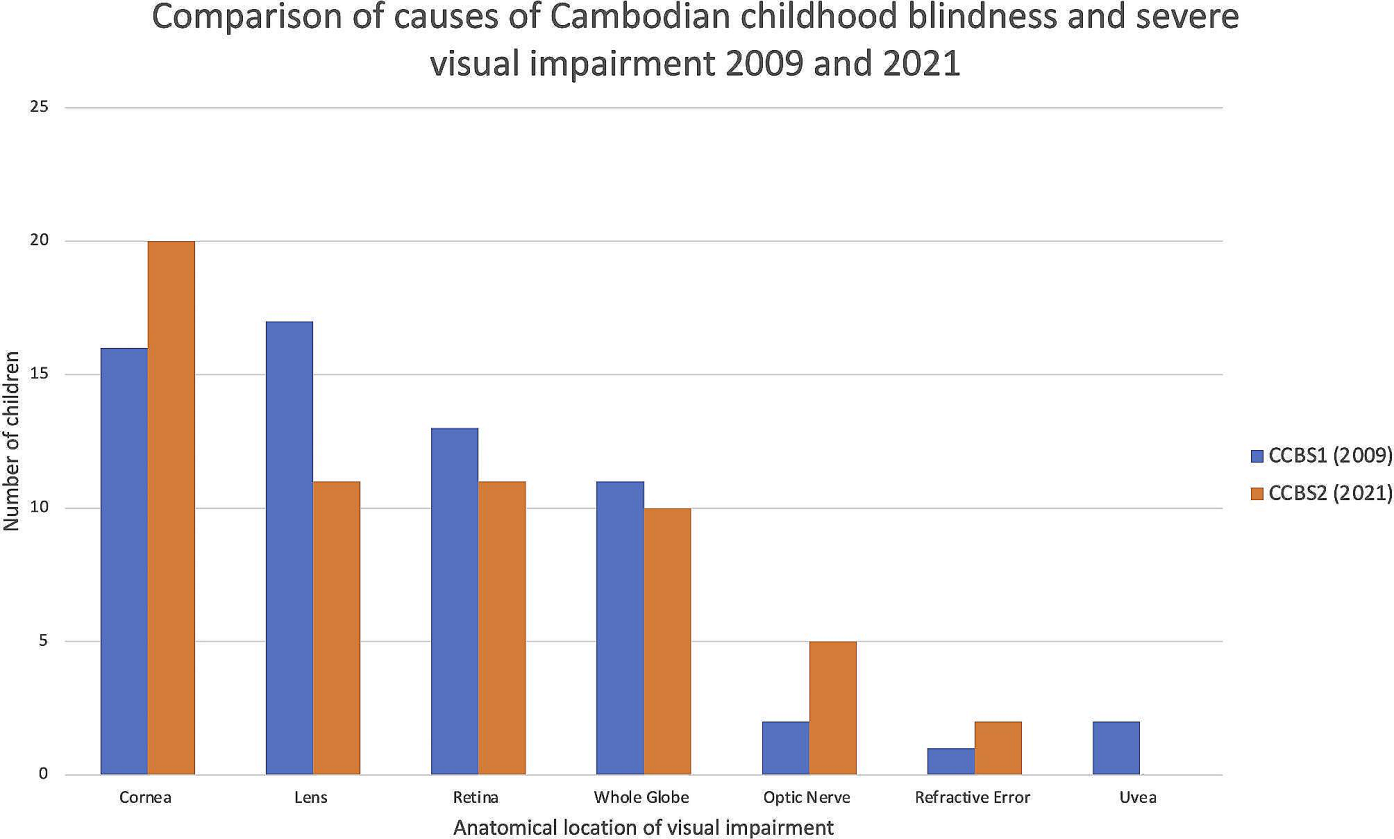
Comparison of causes of Cambodian childhood blindness and severe visual impairment 2009 and 2021 Legend: Column graph showing a comparison between 2009 and 2021 of causes of childhood blindness and severe visual impairment amongst Cambodian children attending schools for the blind

## Discussion

Comparing our findings to the first Cambodian Childhood Blindness Study (CCBS1) we can see that the causes of blindness in Cambodia have not changed significantly since 2009 in spite of the enhanced focus on Vitamin A supplementation and measles immunisation programs which would be expected to decrease avoidable causes of corneal blindness (Table 5; Fig. 1). Additionally, the number of children blind due to lenticular (17 students in 2009 vs. 11 students in 2021) and retinal causes (13 vs. 11 students) was not significantly different between 2009 and 2021. Compared to neighbouring countries, the distribution of causes of eye disease in Cambodian children appears to be similar to those reported in Laos [14] and provincial towns in the Philippines [15] while there is a greater proportion of corneal causes of blindness than that seen in Malaysia [16], Mongolia [7] or Sri Lanka [17].

Arguably, the best studied population detailing the changing causes of childhood blindness as economic conditions improve over time is in India. From these data, it is possible to chart the change in proportionate contributions to childhood blindness from a dominance of corneal pathologies around the turn of the millennium to afflictions of the whole globe and the rise of retinopathy of prematurity (ROP) [6]. Comparing the present study against these data, we see that the current distribution of causes of childhood blindness is similar to that seen in India in studies performed between 1995 and 2007; however, it has yet to undergo the transition towards whole globe and retinal pathologies demonstrated by studies from India over the past decade. The reasons for this are clearly multifactorial, but one obvious contributor is the disparity in economic development between India and Cambodia and the resultant impact this has on the development of health care infrastructure and capabilities [10].

### Childhood blindness and economic correlations

Childhood blindness both begets poverty and is borne of poverty. Previous studies have shown that in addition to being a root cause for the overall prevalence of childhood blindness, the wealth of a country is also a key determinant of the profile of childhood blindness that is likely to predominate [18]. Within this framework, 4 broad categories can be formed to demonstrate a preponderance of corneal causes of childhood blindness amongst very low income countries, lenticular causes in low income countries, retinal causes in middle income countries and disorders of the optic nerve and central nervous system amongst established market economies [18–20]. In addition to this, surveys from China and India have shown that as economies grow over time so too do the proportionate contributions to childhood blindness change with them [6, 21, 22].

CCBS1 and CCBS2 sit astride a period of rapid economic development in Cambodia. Even accounting for the recent economic downturn arising from the coronavirus pandemic, the Cambodian economy has grown at an average rate of 5.9% per annum since CCBS1 was conducted [23, 24]. It is therefore surprising that there is still a such a significant amount of corneal pathology seen in the students from the present study. This is perhaps due to the fact that students in the reported survey had a median age of 13 with 83% of all students becoming blind in the first year of life potentially incurring these blinding conditions before the advantages of contemporary Cambodian healthcare practices had a chance to materialise. However, it is important to note that the growth of an economy does not intrinsically treat childhood blindness and rather it is the new-found ability of these economies to establish primary health education programmes, fund ophthalmic clinics and foster referral pathways that delivers measurable real-world impacts. Based upon the data reported in the present study, further review of the management of corneal causes of childhood blindness and the root risk factors for these such as Vitamin A deficiency, ophthalmia neonatorum, and measles immunisation may be required in Cambodia to reduce this eminently avoidable cause of childhood blindness.

### Local context and interventions

Since 2009, the Cambodian Ministry of Health, National Programme for Eye Health and non-government organisations have introduced a number of interventions with the goal of reducing avoidable causes of childhood blindness. Recent initiatives have included an increase in coverage of measles immunisation and vitamin A supplementation however these attempts have yielded only modest results. The rate of measles immunisation has increased from 76.9% of children aged 12-23months in 2005 to 78.6% in 2014 while the rate of Vitamin A deficiency (which was not reported by the Cambodia Demographic and Health Survey of 2005) has decreased from 42% of children in 2008 to 38.2% of children aged 6-59months being either deficient (9.2%) or marginally deficient (29.2%) [5, 25, 26].

In addition to these medicinal programmes there have also been structural changes to the delivery of ophthalmic services in Cambodia that have led to the commencement of training sub-specialised paediatric ophthalmologists. The advent of this programme is timely, and reflects the development of Cambodian healthcare infrastructure more generally. One interesting feature of the current study is that the 27% of the included children had previously undergone ophthalmic surgery and only 4 students with cataracts had not been operated on. This indicates a growing level of access to paediatric ophthalmic care and may explain why there were not more causes of blindness observed that were attributable to lenticular causes such as congenital cataract. Similarly, although Cambodian neonatal intensive care is still in its naissance, and there is a limited amount of ROP in Cambodia, it is hoped that this resource of paediatric ophthalmologists may serve to screen for and mitigate the eventual rise of ROP that is likely to ensue as Cambodian neonatal care develops.

### Limitations

There are some key limitations of the current study both in general terms related to the selected methodology of a survey of students attending schools for the blind and specific to the study itself. With respect to schools for the blind, in general, these schools do not accept pupils who are below pre-school age or who have multiple disabilities. Further, disadvantaged children from low socioeconomic groups and rural locations are likely to be under-represented in these populations. While both of these factors limit the extent to which data from surveys of schools for the blind can be used to infer information about a nation as a whole, previous authors have shown that with the exception of under-reporting cortical blindness data from these studies are statistically similar to large population based surveys and are a useful data point in assessing nation-wide causes of blindness [6–8].

A further limitation is that children attending schools for the blind were born up to 15 years previously and therefore the conditions seen may not be representative of the current breakdown of new causes of childhood blindness in “real-time”. In view of these limitations, it is fortunate that studies from India, Mongolia, and Uganda have demonstrated that blind children in the community share similar characteristics with blind children attending schools for the blind [6–8]. Further it is important to consider the practical limitations to the “gold-standard” cross-sectional prevalence survey in this field. Such studies require extremely large sample sizes in order to get an accurate depiction of causes of childhood blindness, consequently they are expensive to conduct, and also rely on blind children being present in the community and not away staying with relatives elsewhere or in residential care as is often the case. Conversely, one of the key advantages of a survey of students attending schools for the blind is it enables researchers to identify and review a large number of blind children in a relatively short period of time.

Although it is accurate to say that surveys of students attending schools for the blind are record the causes of blindness with a time lag as the students in attendance often became blind many years previously, the high proportion of students with corneal blindness seen in the present study is representative of the current state of Cambodian ophthalmic public health as the average age of this subgroup of students was 12 years old (SD +/-3.0, range 7–16 years). This means that the average student with corneal blindness was born after the last study of causes of childhood blindness was conducted in 2009 and that they are reflective of the current intervention strategies that were implemented following the prior study.

With respect to the unique conditions faced by our study itself, it was significant that there were 23 children enrolled at schools for the blind who were not included in the present study. It is significant that this study was conducted in the midst of the coronavirus pandemic and prior to the advent of mass COVID-19 immunisation programmes in Cambodia. Of the absent children, 21 were learning from home either due to quarantine requirements or parental choice and 2 were infected with COVID-19. None were excluded due to a refusal to consent school on the day of examination.

## Conclusion and future steps

A striking and surprising finding of the present study was the persistence of corneal causes of vision loss amongst students surveyed at schools for the blind. In the context of general economic development and specific Vitamin A supplementation and measles immunisation programmes it was hypothesised that corneal pathologies would be decreasing in prevalence however this has not been found to be the case with nearly half of all avoidable blindness attributable to corneal disease. Further work is required to understand possible deficiencies in these prevention programmes possibly leveraging the newly established paediatric ophthalmology sub-specialty capabilities to drive demonstrable reductions in avoidable causes of childhood blindness.

## Data Availability

The datasets generated and analysed during the current study are available from Figshare, 10.25909/23538771.v1.

## References

[1] Gilbert C, Foster A (2001). Childhood blindness in the context of VISION 2020–the right to sight. Bull World Health Organ.

[2] Gilbert C (2007). Changing challenges in the control of blindness in children. Eye (Lond).

[3] World Health Organization Blindness and Deafness Unit & International Agency for the Prevention of Blindness. Preventing blindness in children: report of a WHO/IAPB scientific meeting, Hyderabad, India, 13–17 April 1999. World Health Organization; 2000.

[4] World report on vision. Geneva: World Health Organisation; 2019. p. 151.

[5] National Institute of Statistics DGfH, and ICF International. Cambodia Demographic and Health Survey 2014. National Institute of Statistics, Directorate General for Health, and ICF International; 2015.

[6] Wadhwani M, Vashist P, Singh SS, Gupta V, Gupta N, Saxena R (2020). Prevalence and causes of childhood blindness in India: a systematic review. Indian J Ophthalmol.

[7] Bulgan T, Gilbert CE (2002). Prevalence and causes of severe visual impairment and blindness in children in Mongolia. Ophthalmic Epidemiol.

[8] Waddell KM (1998). Childhood blindness and low vision in Uganda. Eye (Lond).

[9] National Institute of Statistics and Ministry of Planning (2019). General Population Census of the Kingdom of Cambodia 2019.

[10] The World Bank. The World Bank Data https://data.worldbank.org/: The World Bank Group; 2022.

[11] Sia DI, Muecke J, Hammerton M, Ngy M, Kong A, Morse A (2010). A survey of visual impairment and blindness in children attending four schools for the blind in Cambodia. Ophthalmic Epidemiol.

[12] blindness. WPftpo. Management of low vision in children: report of a WHO consultation, Bangkok. World Health Organisation. 1993.

[13] Gilbert C, Foster A, Négrel A, Thylefors B (1993). Childhood blindness: a new form for recording causes of visual loss in children. Bull World Health Organ.

[14] Farmer LD, Ng SK, Rudkin A, Craig J, Wangmo D, Tsang H, et al. Causes of severe visual impairment and blindness: comparative data from Bhutanese and Laotian schools for the blind. Asia-Pacific J Ophthalmol (Philadelphia Pa). 2015;4(6):350–6. 10.1097/APO.0000000000000152 26716431

[15] Gilbert C, Foster A (1993). Causes of blindness in children attending four schools for the blind in Thailand and the Philippines. A comparison between urban and rural blind school populations. Int Ophthalmol.

[16] Reddy SC, Tan BC (2001). Causes of childhood blindness in Malaysia: results from a national study of blind school students. Int Ophthalmol.

[17] Eckstein MB, Foster A, Gilbert CE (1995). Causes of childhood blindness in Sri Lanka: results from children attending six schools for the blind. Br J Ophthalmol.

[18] Gilbert C (2007). Changing challenges in the control of blindness in children. Eye (Lond).

[19] Rahi JS, Sripathi S, Gilbert CE, Foster A (1995). Childhood blindness due to vitamin A deficiency in India: Regional variations. Arch Dis Child.

[20] Kemmanu V, Hegde K, Giliyar SK, Shetty BK, Kumaramanickavel G, McCarty CA (2016). Prevalence of Childhood blindness and ocular morbidity in a rural Pediatric Population in Southern India: the Pavagada Pediatric Eye Disease Study-1. Ophthalmic Epidemiol.

[21] Hornby SJ, Xiao Y, Gilbert CE, Foster A, Wang X, Liang X (1999). Causes of childhood blindness in the People’s Republic of China: results from 1131 blind school students in 18 provinces. Br J Ophthalmol.

[22] Li Y, Yan J, Wang Z, Huang W, Huang S, Jin L (2019). Prevalence and causes of childhood blindness in Huidong County, South China, primary ascertained by the key informants. BMJ Open Ophthalmol.

[23] Fund IM. International Monetary Fund - Cambodia Country Data https://www.imf.org/en/Countries/KHM#whatsnew: International Monetary Fund; 2021 [updated 2021. Available from: https://www.imf.org/en/Countries/KHM#whatsnew

[24] Fund IM, Staff CC (2019). CAMBODIA– 2019 ARTICLE IV CONSULTATION.

[25] Chakrya KS. Nearly half of Cambodian children under 5 lack vitamin A. Phnom Penh Post; 2008.

[26] National Institute of Public Health National Institute of Statistics (Cambodia) and ORC Macro (2006). Cambodia Demographic and Health Survey 2005. Phnom Penh, Cambodia and Calverton.

